# A GATE simulation study for dosimetry in cancer cell and micrometastasis from the ^225^Ac decay chain

**DOI:** 10.1186/s40658-023-00564-5

**Published:** 2023-08-01

**Authors:** Helena Koniar, Cassandra Miller, Arman Rahmim, Paul Schaffer, Carlos Uribe

**Affiliations:** 1 Life Sciences Division, TRIUMF, Vancouver, BC Canada; 2 Department of Physics and Astronomy, University of British Columbia, Vancouver, BC Canada; 3 Department of Integrative Oncology, BC Cancer Research Institute, Vancouver, BC Canada; 4 Department of Radiology, University of British Columbia, Vancouver, BC Canada; 5 Department of Chemistry, Simon Fraser University, Burnaby, BC Canada; 6 Functional Imaging, BC Cancer, Vancouver, BC Canada

**Keywords:** Actinium-225, Radiopharmaceutical therapy, Microdosimetry, Cellular *S* value, Monte Carlo simulations

## Abstract

**Background:**

Radiopharmaceutical therapy (RPT) with alpha-emitting radionuclides has shown great promise in treating metastatic cancers. The successive emission of four alpha particles in the ^225^Ac decay chain leads to highly targeted and effective cancer cell death. Quantifying cellular dosimetry for ^225^Ac RPT is essential for predicting cell survival and therapeutic success. However, the leading assumption that all ^225^Ac progeny remain localized at their target sites likely overestimates the absorbed dose to cancer cells. To address limitations in existing semi-analytic approaches, this work evaluates *S*-values for ^225^Ac’s progeny radionuclides with GATE Monte Carlo simulations.

**Methods:**

The cellular geometries considered were an individual cell (10 µm diameter with a nucleus of 8 µm diameter) and a cluster of cells (micrometastasis) with radionuclides localized in four subcellular regions: cell membrane, cytoplasm, nucleus, or whole cell. The absorbed dose to the cell nucleus was scored, and self- and cross-dose *S*-values were derived. We also evaluated the total absorbed dose with various degrees of radiopharmaceutical internalization and retention of the progeny radionuclides ^221^Fr (*t*_1/2_ = 4.80 m) and ^213^Bi (*t*_1/2_ = 45.6 m).

**Results:**

For the cumulative ^225^Ac decay chain, our self- and cross-dose nuclear *S*-values were both in good agreement with *S*-values published by MIRDcell, with per cent differences ranging from − 2.7 to − 8.7% for the various radionuclide source locations. Source location had greater effects on self-dose *S*-values than the intercellular cross-dose *S*-values. Cumulative ^225^Ac decay chain self-dose *S*-values increased from 0.167 to 0.364 GyBq^−1^ s^−1^ with radionuclide internalization from the cell surface into the cell. When progeny migration from the target site was modelled, the cumulative self-dose *S*-values to the cell nucleus decreased by up to 71% and 21% for ^221^Fr and ^213^Bi retention, respectively.

**Conclusions:**

Our GATE Monte Carlo simulations resulted in cellular *S*-values in agreement with existing MIRD *S*-values for the alpha-emitting radionuclides in the ^225^Ac decay chain. To obtain accurate absorbed dose estimates in ^225^Ac studies, accurate understanding of daughter migration is critical for optimized injected activities. Future work will investigate other novel preclinical alpha-emitting radionuclides to evaluate therapeutic potency and explore realistic cellular geometries corresponding to targeted cancer cell lines.

## Background

Targeted radiopharmaceutical therapy (RPT) involves using antigen-targeted molecules that are conjugated with alpha, beta, or Auger-electron emitting radionuclides, injected to accumulate, irradiate, and selectively kill cancer cells while sparing surrounding non-target tissue [1]. The therapeutic application of alpha emitters is of interest due to their short range and high energy deposition that results in effective cell death of the targeted cells and minimal damage to neighbouring normal healthy tissue [1]. Therapies with alpha particles have two distinct advantages over beta- or Auger-RPTs. First, the short range of alpha radiation in human tissues corresponds to only a few cell diameters (< 0.1 mm) where DNA damage can occur [2]. Second, the high energy (~ 5 MeV) and high linear energy transfer (LET) (~ 100 keV/µm) of alpha emissions leads to highly effective cell killing via DNA double strand breaks [2]. Because alpha particles deliver highly cytotoxic radiation with a limited range, they are ideal for the treatment of smaller tumour burdens, micrometastatic disease, and disseminated disease while avoiding damage to non-targeted cells [3].

**Fig. 1:**
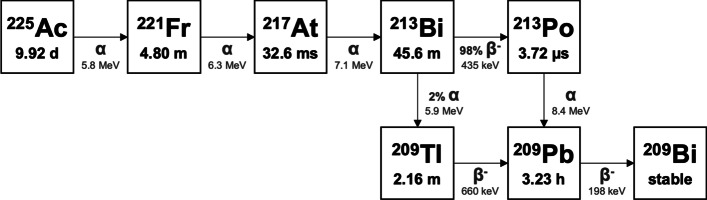
^225^Ac decay scheme to stable ^209^Bi

Actinium-225 (^225^Ac) is a promising candidate isotope for RPT due to it successive cascade of high energy emissions with a total alpha energy of 27.6 MeV throughout its decay series and its relatively long-lived half-life of 9.92 days [4]. ^225^Ac has also been validated as a therapeutic radionuclide in several preclinical human studies [5]. This isotope has been targeted against many cancers including prostate, leukaemia, glioma, neuroendocrine, and melanoma, with remarkable outcomes in tumour control [6–13]. ^225^Ac decays to stable ^209^Bi through six short-lived radionuclide daughters (see Fig. 1). The decay cascade of ^225^Ac yields four alpha particles, two beta particles, and two gamma emissions. Specifically, ^225^Ac (*t*_1/2_ = 9.92 d; 5.8 MeV α-particle) decays to ^221^Fr (*t*_1/2_ = 4.80 m; 6.3 MeV α-particle), ^217^At (*t*_1/2_ = 32.6 ms; 7.1 MeV α-particle), ^213^Bi (*t*_1/2_ = 45.6 m; 435 keV mean energy β^−^-particle with 97.8% branching ratio or 5.9 MeV α-particle with 2.2% branching ratio), ^213^Po (*t*_1/2_ = 3.72 μs; 8.4 MeV α-particle), ^209^Tl (*t*_1/2_ = 2.16 m; 660 keV mean energy β^−^-particle), ^209^Pb (*t*_1/2_ = 3.23 h; 198 keV mean energy β^−^-particle) and ^209^Bi (stable). The four alpha-particles have energies ranging from 5.8 to 8.4 MeV with associated tissue ranges of 47 to 85 µm [14]. Additionally, the two β^−^-particles from the decay chain have therapeutic effects at further physical distances than the alpha emissions. The gamma emissions throughout the ^225^Ac decay chain can be used to assess biodistribution through imaging from the decay of ^221^Fr (218 keV, 11.6% branching ratio) and ^213^Bi (440 keV, 26.1% branching ratio) [15, 16].

In the context of RPT with an alpha emitter, internal radiation dosimetry is necessary for several reasons: first, to understand the radiobiological response; second, to compare and evaluate the efficacy of various preclinical therapeutic radionuclides; and third, to optimize the administered activity to achieve a personalized treatment. The standard method for calculating radiation dose from administered activity has been formalized by the Medical Internal Radiation Dosimetry (MIRD) schema which is applicable at organ, sub-organ, voxel, multicellular, and cellular levels [17]. The *S*-value defines the mean absorbed dose to a target region per radionuclide decay in a source region [18]. The absorbed dose is thus a product of the cumulative radioactivity over a time of interest and the *S*-value. When targeting individual cells and small tumour metastases, the dose to individual cells becomes important as the range of the emitted alpha particles is comparable in scale to the diameter of a cell or a small cluster of cells. Comprehensive databases of cellular *S-*values for many radionuclides of interest with spherical cells of various sizes and radionuclide distributions have been published by MIRD [19].

The calculations of cellular *S*-values by MIRD are based on convolution integrals, a semi-analytic method which applies the continuous slowing down approximation (CSDA). To address the limitations of the CSDA methodology, Monte Carlo track-structure (MCTS) methods have been studied to evaluate a number of radionuclides at a cellular level via simulations of radiation transport [18, 20–29]. However, in most studies, the focus has been largely on beta-emitting radionuclides with simple decay schemes, while a limited number of dosimetry studies focused on alpha emitters or ^225^Ac, specifically [22, 30, 31].

With respect to ^225^Ac and its complex decay chain, it is important to quantify the absorbed dose contributions from each radionuclide in the decay chain to determine the effect that progeny migration from the targeted cell will have on the overall therapeutic dose. During alpha decay, the recoil typically imparts an energy of ~ 0.1 MeV to the daughter nuclide, which is orders of magnitude higher than a typical chemical binding energy. The result is a daughter radionuclide that is no longer chemically bound to the radiopharmaceutical complex. The transfer of daughter radiation dose to off-target sites depends on its half-life, diffusion properties, and affinity for organs at risk. The first progeny in the decay of ^225^Ac is ^221^Fr (*t*_1/2_ = 4.80 m), which is a potassium analogue and is excreted from the cell via Na^+^/K^+^ pumps, potentiating subsequent decays occurring outside the target cells. The longer lived ^213^Bi (*t*_1/2_ = 45.6 m) can migrate to the kidneys, which may cause renal toxicity [32, 33]. There are many efforts underway to mitigate daughter migration from the target site, including targeting rapid cellular internalization, localized administration, and encapsulations in nanoparticles [34]. However, for current ^225^Ac-based therapies, daughter migration is often the limiting factor on injected activity and, consequently, therapeutic dose [34–36].

Here, we apply a MCTS approach to alpha emitters and, more importantly, to the complex ^225^Ac decay chain to discern the effects of radiopharmaceutical internalization and daughter migration. In this in silico study, we aim at evaluating the absorbed dose for radionuclides in the ^225^Ac decay chain (^225^Ac, ^221^Fr, ^217^At, ^213^Bi, ^213^Po, ^209^Tl, and ^209^Pb) for an individual cell and a cellular cluster to represent a micrometastatic tumour. To account for different levels of internalization of a radiopharmaceutical, we characterize the intra- and intercellular absorbed dose depositions to the cell nucleus for various subcellular distributions. We analyse the self- and cross- dose contributions. The dependency of *S*-values on the degree of radiopharmaceutical internalization and daughter radionuclide retention is also evaluated. The data presented here can assist in dosimetry calculations for novel ^225^Ac radiopharmaceuticals as they are evaluated in preclinical settings.

## Methods

### Monte Carlo simulations in GATE

We computed absorbed doses with simulations of radiation tracks in matter using the Monte Carlo simulations software GATE (Geant4 Application for Tomographic Emission) version 9.0. GATE uses physics modelling based on Geant4 (GEometry ANd Tracking) version 10.6.1 [37]. This toolkit was selected because of its well validated features for internal dosimetry in nuclear medicine [38–40].

All simulations used the low energy extended electromagnetic physics list *emDNAphysics* based on G4EmDNAPhysics (default constructor) of Geant4-DNA. The physical models loaded include all relevant electromagnetic physics processes, the interactions of all generated particles, and associated models required for the simulations. It enables collision-by-collision simulation of electron tracks down to the excitation threshold of liquid water (7.4 eV). We used the Geant4-DNA physics list because of its suitability for microdosimetry applications [41, 42] and good agreement with data obtained using other Monte Carlo codes like CELLDOSE, MC4V, PENELOPE, MCNP, and EGSnrc [27].

### Modelled cellular geometries

**Fig. 2:**
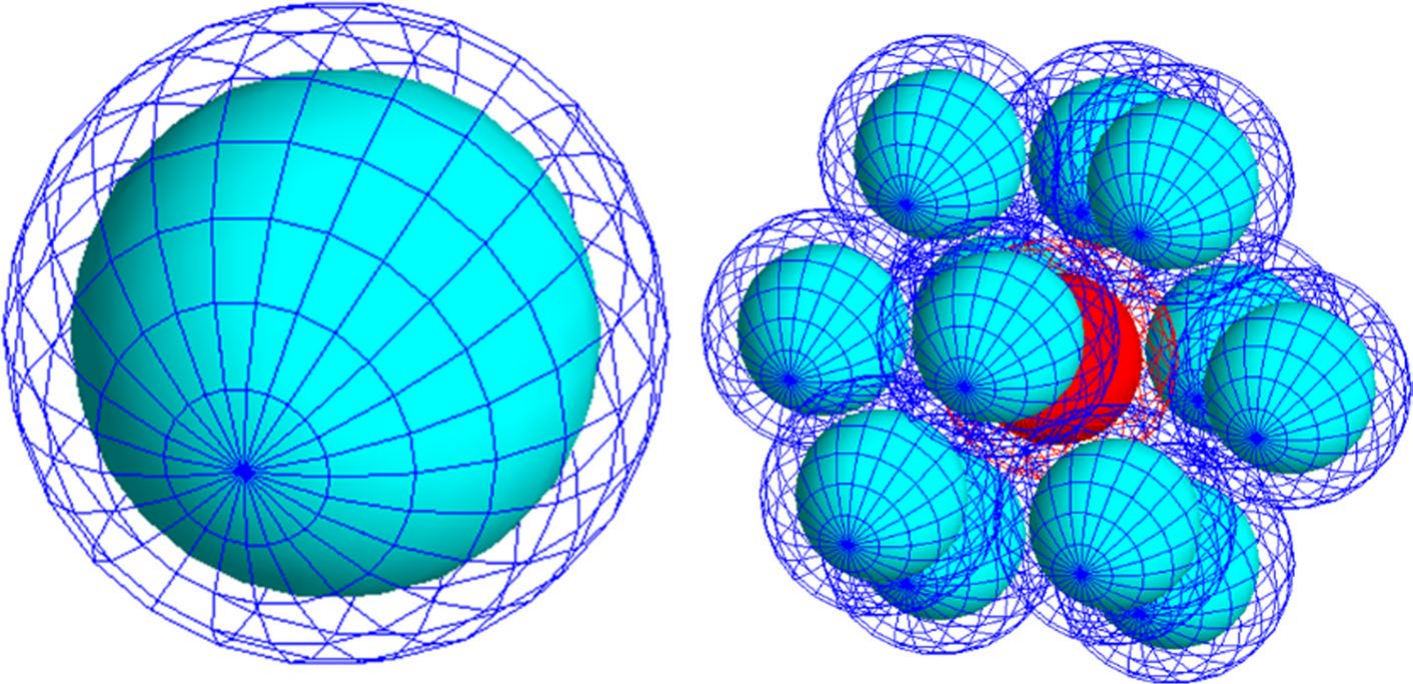
(left) Single cell of 10 µm diameter with a nucleus of 8 µm diameter. (right) Cell cluster modelled in this work. Dark blue represents the cell membrane and light blue represents the cell nucleus

We considered two cellular geometries: an individual cell and a cluster of cells that represent a micrometastasis. The single cell model consists of two concentric spheres representing the cell and the cell nucleus. The cell has a 10 µm diameter while the nucleus has an 8 µm diameter (See Fig. 2a). This geometry is based on formalized MIRD models and previous studies, to allow for direct comparison to previously established *S*-values [18, 27, 28]. The micrometastasis model consists of a cluster of individual cells (see Fig. 2b). Previous studies have considered a simple cubic structure model; however, we considered a hexagonal lattice as this provides more biologically reasonable spacing [43, 44]. Following a 3D hexagonal close packed lattice, the cluster consisted of a central cell with 12 neighbouring cells that are equidistance from the central cell. The central and neighbouring cells are simulated with the same cellular geometry as the isolated cell described previously. The material used for all cellular components was *G4_WATER* which is included in the GATE material database with a density of 1 g/cm^3^. While this cellular geometry simplifies the true in vivo structure of cancer cells and micrometastasis, it facilitates direct comparison to existing values derived from a semi-analytic method to establish the validity of Geant4-DNA Monte Carlo simulations for more interesting realistic cellular geometries in future works.

### ^225^Ac decay chain

We individually simulated the decays of each radionuclide in the ^225^Ac decay scheme (see Fig. 1) to quantify the absorbed dose contributions with respect to each nuclide decay. Each radionuclide of interest is uniformly distributed in one of the following regions of the cell: (i) only on the cell membrane, (ii) only in the cytoplasm, (iii) only in the cell nucleus, or (iv) throughout the whole cell. There is equal radionuclide distribution in all cells of the cluster. GATE’s Radioactive Decay Module (RDM) simulates radioactive decays with secondary particle emissions on a per decay level [45, 46]. RDM produces primary particles using data from the evaluated nuclear structure data file (ENSDF) including branching ratios, decay energies and transition probabilities, and energies for nuclear de-excitation. RDM simulates the decay of radioactive nuclei through α, β^−^, β^+^, isomeric transition, and electron capture. RDM simulates the entire decay and its associated decay emissions. With the GATE *particleFilter* tool, we can filter the atomic number (*Z*) and mass number (*A*) of the daughter progeny to stop the decay chain from continuing. The *KillActor* tool removes the daughter progeny from the simulation, so that we can simulate the dose deposition from each radionuclide individually.

The radionuclide source was input with source definitions (^225^Ac, ^221^Fr, ^218^At, ^213^Bi, ^213^Po, ^209^Tl, and ^209^Pb) and source distributions, or source location, (cell membrane, cytoplasm, nucleus, and whole cell). In all simulations, the total number of primary particles simulated was defined as 100,000 for alpha-emitting radionuclides (^225^Ac, ^221^Fr, ^218^At, ^213^Po) and 2,000,000 for beta-emitting radionuclides (^213^Bi, ^209^Tl, and ^209^Pb). Each combination of radionuclide and source distribution was simulated with Mersenne Twister random number generator [47] with each combination split into 10 separate jobs to reduce total run time. The number of primaries for alpha versus beta emission was determined to achieve comparable counting statistics, low uncertainties in the absorbed dose, and attainable simulation run times.

### Measuring the absorbed dose

The GATE *DoseActor* tool scored the absorbed dose to each voxel, and its associated statistical uncertainty. This information is stored in a 3D matrix, or image, which enables fast visualization of the dose distribution. In our simulations, we scored the absorbed dose only to the nucleus, as it is the primary target for radiation-induced cell death [48]. For the cluster simulations, we scored the absorbed dose to the nucleus of the central cell, denoted in red in Fig. 2b. Given the geometrical symmetry of the cluster, the dose absorbed in the central cell is equally contributed from all 12 nearest neighbouring cells. Our voxel resolution was set to a matrix size of 100 × 100 × 100, with an individual voxel with dimensions of 0.8 µm × 0.8 µm × 0.8 µm.

The output of each simulation was a 3D matrix file containing the dose to each voxel. The dose volumetric data were analysed with Python v3.8.8 to calculate the total dose and associated uncertainties from the generated output files. The dose output images from each run were averaged together, and the total absorbed dose to the nucleus $(D_{{{\text{nuc}}}} )$ was calculated by the mean value of dose in each voxel in the region.

$$D_{{{\text{nuc}}}} = \frac{{E_{{{\text{tot}}}} }}{{M_{{{\text{nuc}}}} }} = \frac{{\sum E_{{{\text{vox}}}} }}{{M_{{{\text{nuc}}}} }} = \frac{{\sum E_{{{\text{vox}}}} }}{{n m_{{{\text{vox}}}} }} = \frac{{\sum D_{{{\text{vox}}}} }}{n}$$

where $E_{{{\text{tot}}}}$ is the total energy deposited in the nucleus, $M_{{{\text{nuc}}}}$ is the total mass of the nucleus, $E_{{{\text{vox}}}}$ is the energy deposited in an individual voxel, $m_{{{\text{vox}}}}$ is the mass of an individual voxel, and $n$ is the number of voxels in the nucleus region. GATE also scores the dose squared in each voxel which is used to calculate the uncertainty with several runs. The standard error in the mean dose in each voxel $\left( {u\left[ {D_{{{\text{vox}}}} } \right]} \right)$ was calculated according to the following equation [49].

$$u\left[ {D_{{{\text{vox}}}} } \right] = \sqrt {\frac{1}{N - 1}\left( {\frac{{\sum D_{{{\text{vox}}}}^{2} }}{N} - \left( {\frac{{\sum D_{{{\text{vox}}}} }}{N}} \right)^{2} } \right)}$$

where $N$ is the number of primaries simulated. The standard error in the dose to the nucleus ($u\left[ {D_{{{\text{nuc}}}} } \right]$) is calculated with the following equation.

$$u\left[ {D_{{{\text{nuc}}}} } \right] = \frac{{\sum u[D_{{{\text{vox}}}} ]}}{n}$$

The number of primaries simulated ensured that the relative uncertainty in total dose is < 0.5% for all simulations.

### *S*-value calculations

According to the MIRD scheme, the mean absorbed dose in a target region ($r_{{\text{T}}}$), $D\left( {r_{{\text{T}}} \leftarrow r_{{\text{S}}} } \right)$, due to the cumulated activity, $A_{{\text{C}}}$, in a source region ($r_{{\text{S}}}$) is defined by the following formula:

$$D\left( {r_{{\text{T}}} \leftarrow r_{{\text{S}}} } \right) = A_{{\text{C}}} \times S\left( {r_{{\text{T}}} \leftarrow r_{{\text{S}}} } \right)$$

where $S\left( {r_{{\text{T}}} \leftarrow r_{{\text{S}}} } \right)$, is known as the *S*-value and denotes the dose to the target region per decay in the source region [19]. The *S*-values were calculated in units of GyBq^−1^ s^−1^, by dividing the total dose by the number of primary particles simulated.

We calculated the self-dose *S*-values directly from the individual cell simulations and calculated the cross-dose *S*-values from the cluster simulations. When considering the absorbed dose to the centre cell in a micrometastasis, it receives self-dose, from radionuclides localized within the central cell, and cross-dose, from radionuclides localized within all neighbouring cells [50]. Thus, the self-dose *S*-value is needed to derive the cross-dose *S*-value from absorbed dose to the centre cell’s nucleus. The ‘total’ *S*-values for self- and cross-dose were also calculated by summing the dose contributions from all radionuclides in the ^225^Ac decay chain, with consideration for branching ratios (see Fig. 1). The cumulative *S*-values represent the dose absorbed from the decay of ^225^Ac with the assumption that all progeny nuclides stay localized to the initial decay site. Cellular *S*-values were compared to the values from MIRDcell v3.10 software (Rutgers New Jersey Medical School, Newark, NJ) [51].

### Radionuclide retention and internalization

By individually simulating each radionuclide in the ^225^Ac decay chain, we can assess the effect of progeny retention. ^221^Fr (*t*_1/2_ = 4.8 m) and ^213^Bi (*t*_1/2_ = 45.6 m) are the progeny nuclides of most concern when considering in vivo redistribution since they contribute successive alpha emissions following the decay of ^225^Ac. Although ^217^At (*t*_1/2_ = 32.6 ms) and ^213^Po (*t*_1/2_ = 3.72 μs) are also alpha emitters, they have very short half-lives and can be approximated to deposit their dose in the same location as their parent nuclide. ^209^Pb (*t*_1/2_ = 3.23 h) has a longer half-life; however, since it is a beta emitter, it is less critical to the overall therapeutic dose and off-target toxicities. We calculated the total self-dose *S*-values as a function of ^221^Fr and ^213^Bi retention by varying the progeny dose contribution for each source distribution. We considered progeny retention as ranging from 100% (^221^Fr + progeny or ^213^Bi + progeny localized) to 0% (^221^Fr + progeny or ^213^Bi + progeny migrated) in 20% increments.

By simulating various subcellular localization of the radionuclide, we can assess the effect that radiopharmaceutical internalization has on the absorbed dose to the nucleus. We defined cellular internalization as the fraction of radionuclides fully internalized throughout the whole cell relative to radionuclides not internalized and bound to the cellular membrane. We calculated the total self-dose *S*-values as a function of cellular internalization, ranging from 0% (all radionuclides bound to cellular membrane) to 100% (all radionuclides internalized inside the cell) in 20% increments.

## Results

### Self-dose *S*-values

**Fig. 3:**
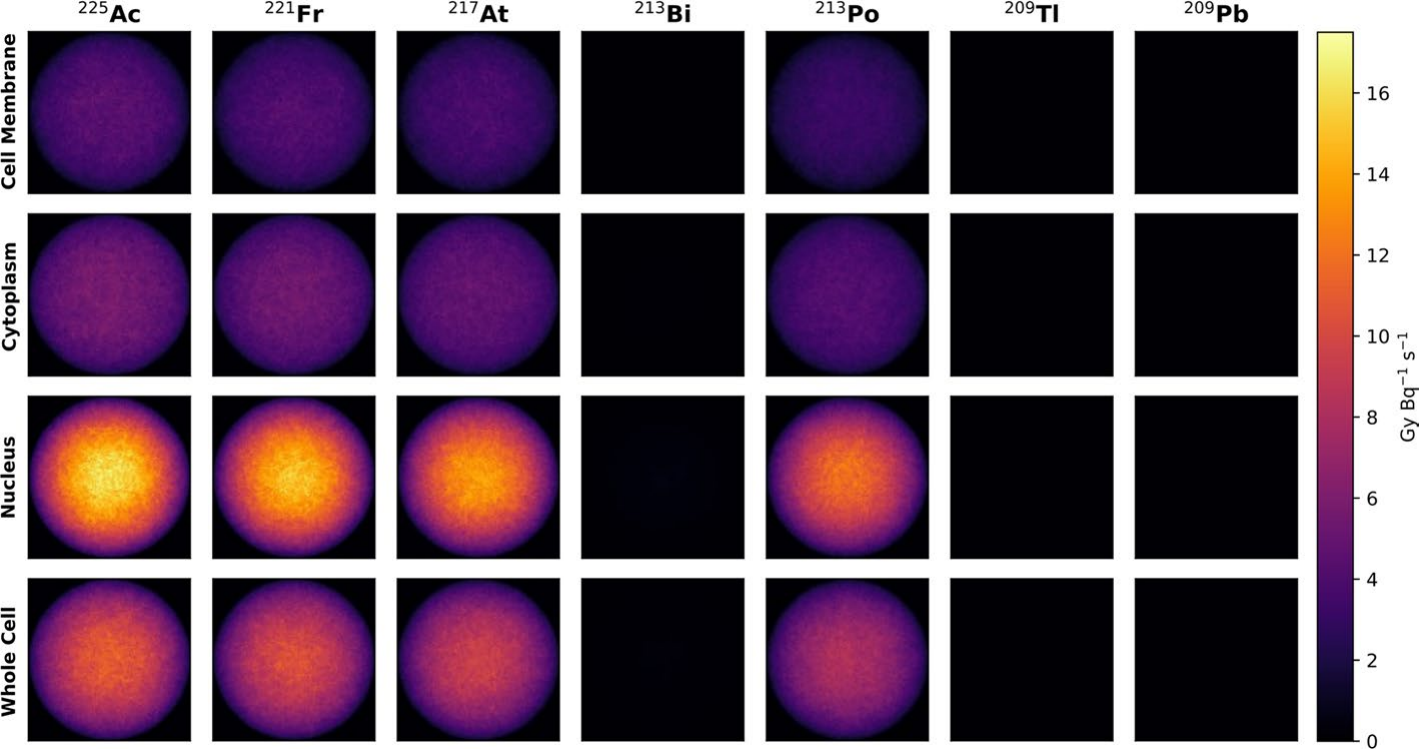
Cell nucleus dose maps for progeny radionuclides in the ^225^Ac decay chain. Radionuclide localization is defined as either bound to the cell membrane, within the cytoplasm, within the nucleus, or throughout the whole cell and the absorbed dose to the cell nucleus is scored. (*Note*: Plots have been normalized to number of primaries simulated and are visualized with equivalent heat map scaling)

**Fig. 4:**
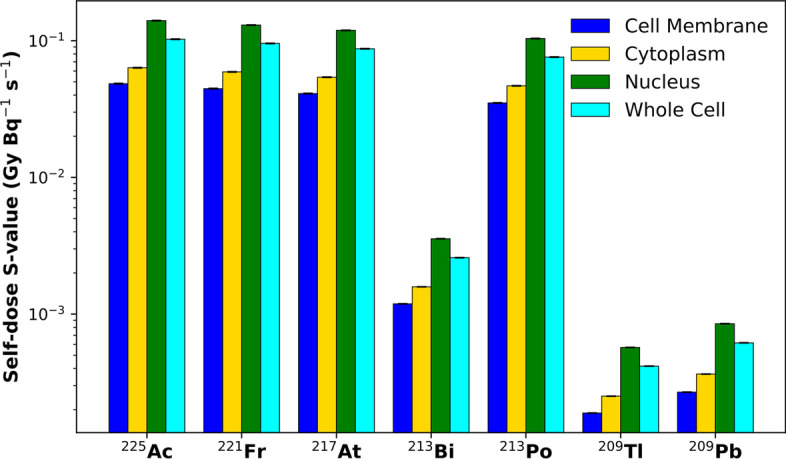
Self-dose *S*-value (GyBq^−1^ s^−1^) to the cell nucleus for ^225^Ac progeny radionuclides. The figure legend indicates the radionuclide’s localization as either bound to the cell membrane (blue), within the cytoplasm (yellow), within the cell nucleus (green), or throughout the whole cell (cyan). Error bars represent one standard error, all with relative uncertainty values < 0.5%. *Note*: the *S*-values have been represented on a logarithmic scale to display the absorbed dose contributions of both alpha- and beta-emitting radionuclides in the ^225^Ac decay chain

Table 1 shows the self-dose *S*-values of the ^225^Ac decay chain radionuclides, as well as a cumulative total for the entire ^225^Ac decay chain with appropriate consideration of branching ratios. Making intuitive sense, the *S*-values are lowest when the radionuclide is localized to the cell surface, and conversely, are highest when internalized within the nucleus (see Fig. 3). Throughout the ^225^Ac decay chain, alpha emissions contribute more absorbed dose than beta emissions (see Fig. 4). For the radionuclides decaying via alpha emission (^225^Ac, ^221^Fr, ^217^At, ^213^Po), there was good agreement between MIRDcell *S*-values, with percentage differences < − 12.1%. However, for the radionuclides which decayed via beta emissions (^213^Bi, ^209^Tl, ^209^Pb), the obtained *S*-values were significantly different from MIRDcell (up to − 70.9% for ^209^Tl). Overall, the total decay chain *S*-values obtained using Geant4-DNA physics were in good agreement with MIRDcell *S*-values with the per cent difference ranging from − 4.7 to − 6.9%.

**Table 1:** Intracellular self-dose *S*-values (GyBq^−1^ s^−1^) for nuclides in the ^225^Ac decay chain from different source distributions targeting the nucleus

Nuclide	*S*-value (GyBq^−1^ s^−1^)			
	Cell surface	Cytoplasm	Nucleus	Whole Cell
^225^Ac	4.84 × 10^–2^ (− 4.4%)	6.29 × 10^–2^ (− 5.9%)	1.40 × 10^–1^ (− 4.5%)	1.02 × 10^–1^ (− 5.1%)
^221^Fr	4.36 × 10^–2^ (− 7.3%)	5.87 × 10^–2^ (− 5.7%)	1.30 × 10^–1^ (− 4.8%)	9.64 × 10^–2^ (− 3.6%)
^217^At	4.09 × 10^–2^ (− 4.9%)	5.42 × 10^–2^ (− 4.8%)	1.18 × 10^–1^ (− 5.5%)	8.73 × 10^–2^ (− 4.8%)
^213^Bi	1.22 × 10^–3^ (− 3.6%)	1.49 × 10^–3^ (− 12.2%)	3.62 × 10^–3^ (− 6.4%)	2.64 × 10^–3^ (− 6.0%)
^213^Po	3.31 × 10^–2^ (− 12.1%)	4.48 × 10^–2^ (− 10.1%)	1.04 × 10^–1^ (− 5.6%)	7.62 × 10^–2^ (− 5.5%)
^209^Tl	1.91 × 10^–4^ (− 48.8%)	2.48 × 10^–4^ (− 56.2%)	5.62 × 10^–4^ (− 70.9%)	4.13 × 10^–4^ (− 67.3%)
^209^Pb	2.70 × 10^–4^ (8.5%)	3.69 × 10^–4^ (8.8%)	8.51 × 10^–4^ (8.1%)	6.17 × 10^–4^ (8.6%)
Total	1.67 × 10^–1^ (− 6.9%)	2.21 × 10^–1^ (− 6.5%)	4.94 × 10^–1^ (− 5.0%)	3.64 × 10^–1^ (− 4.7%)

The numbers in the parentheses represent the per cent differences with respect to MIRDcell reported *S*-values [52]

### Cross-dose *S*-values

**Fig. 5:**
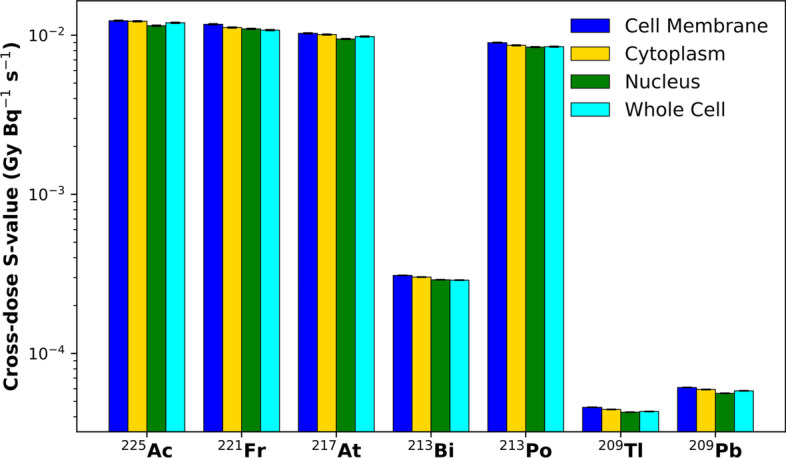
Cross-dose *S*-value (GyBq^−1^ s^−1^) to the cell nucleus of a nearest neighbouring cell for ^225^Ac progeny radionuclides. The figure legend indicates the radionuclide’s localization as either bound to the cell membrane (blue), within the cytoplasm (yellow), within the cell nucleus (green), or throughout the whole cell (cyan). Error bars represent one standard error, all with relative uncertainty values < 0.5%. *Note*: the *S*-values have been represented on a logarithmic scale to display the absorbed dose contributions of both alpha- and beta-emitting radionuclides in the ^225^Ac decay chain

Table 2 shows the intercellular cross-dose *S*-values delivered by the ^225^Ac decay chain radionuclides, as well as a cumulative total for the entire ^225^Ac decay chain, to the nucleus of the nearest neighbouring cell. The cross-dose *S*-values are relatively independent of subcellular localization (see Fig. 5) and primarily depend on radiation properties, such as mean alpha or beta emission energy. Comparing the cross-dose *S*-values obtained with Geant4-DNA physics to MIRDcell *S-*values saw good agreement. The alpha-emitting radionuclides (^225^Ac, ^221^Fr, ^217^At, ^213^Po) are within < 10.9%. However, like the self-dose *S*-value results, the beta-emitting radionuclides (^213^Bi,^209^Tl, ^209^Pb) have larger deviations from the MIRDcell *S*-values, up to − 46.8% for ^209^Tl. Overall, the combined cross-dose *S*-values for the entire decay chain differ between 2.7 and 8.7% from their MIRDcell counterparts.

**Table 2:** Intercellular cross-dose *S*-values (GyBq^−1^ s^−1^) for radionuclides in the ^225^Ac decay chain from different source distributions targeting the cell nucleus of a neighbouring cell

Radionuclide	*S*-value (GyBq^−1^ s^−1^)			
	Cell surface	Cytoplasm	Nucleus	Whole cell
^225^Ac	1.29 × 10^–2^ (− 1.8%)	1.21 × 10^–2^ (− 5.3%)	1.10 × 10^–2^ (− 9.7%)	1.19 × 10^–2^ (− 4.9%)
^221^Fr	1.21 × 10^–2^ (− 0.3%)	1.16 × 10^–2^ (− 1.9%)	1.04 × 10^–2^ (− 7.3%)	1.02 × 10^–2^ (− 10.9%)
^217^At	1.01 × 10^–2^ (− 7.9%)	1.00 × 10^–2^ (− 6.3%)	9.70 × 10^–3^ (− 5.8%)	9.37 × 10^–3^ (− 10.7%)
^213^Bi	3.34 × 10^–4^ (3.5%)	2.75 × 10^–4^ (− 12.7%)	2.77 × 10^–4^ (− 7.5%)	2.84 × 10^–4^ (− 7.5%)
^213^Po	9.38 × 10^–3^ (− 1.5%)	8.91 × 10^–3^ (− 4.2%)	8.23 × 10^–3^ (− 6.7%)	8.25 × 10^–3^ (− 8.9%)
^209^Tl	4.66 × 10^–5^ (− 44.3%)	4.29 × 10^–5^ (− 46.8%)	4.47 × 10^–5^ (− 40.1%)	4.43 × 10^–5^ (− 44.6%)
^209^Pb	6.27 × 10^–5^ (8.3%)	6.07 × 10^–5^ (7.8%)	5.72 × 10^–5^ (8.0%)	5.77 × 10^–5^ (5.7%)
Total	4.46 × 10^–2^ (− 2.7%)	4.28 × 10^–2^ (− 4.5%)	3.95 × 10^–2^ (− 7.5%)	3.99 × 10^–2^ (− 8.7%)

The numbers in the parentheses represent the per cent differences with respect to MIRDcell reported *S*-values [52]

### Radionuclide retention and internalization

**Fig. 6:**
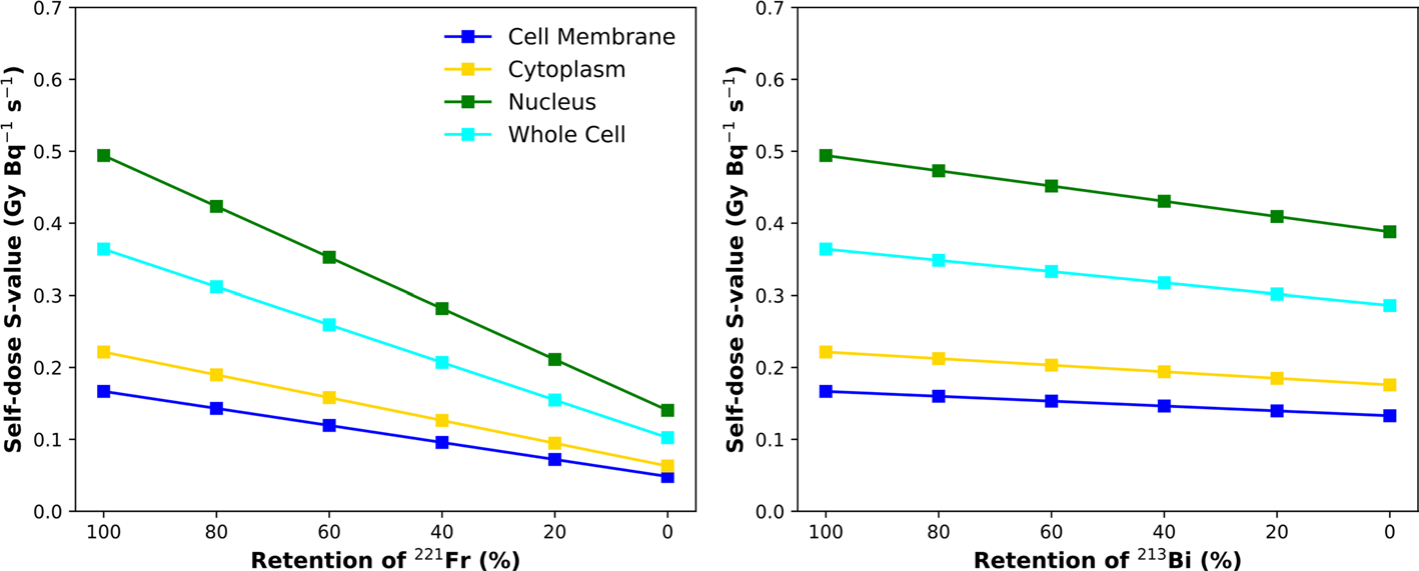
Total self-dose *S*-value (GyBq^−1^ s^−1^) to the cell nucleus with various source distributions as a function of ^221^Fr (left) and ^213^Bi (right) retention

The effects of ^221^Fr and ^213^Bi retention on the total self-dose *S*-values to the cell nucleus are seen in Fig. 6.

**Fig. 7:**
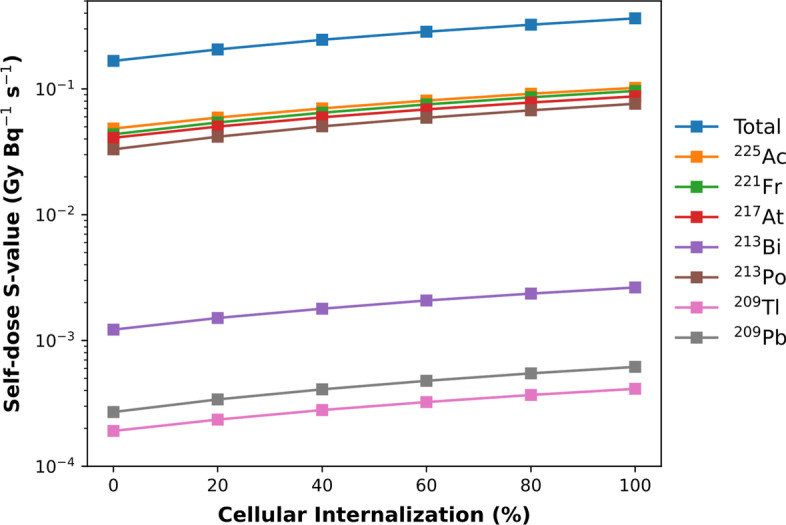
Total self-dose *S*-value (GyBq^−1^ s^−1^) to the cell nucleus with as a function of cellular internalization. *Note*: the *S*-values have been represented on a logarithmic scale to display the absorbed dose contributions of both alpha- and beta-emitting radionuclides in the ^225^Ac decay chain

Figure 7 shows the effect of cellular internalization on the *S-*values for ^225^Ac’s progeny radionuclides from cell membrane localization to whole cell localization. The *S-*values all increase with increasing cellular internalization, by as much as 30% for the alpha emitter ^213^Po.

## Discussion

RPT with alpha emitters has shown remarkable promise as an effective cancer treatment. ^225^Ac’s therapeutic efficacy comes from the four alpha emissions throughout its decay chain. Characterizing the dose delivered within a cancer cell is critical to understanding its therapeutic outcome. The *S*-value metric is a universal parameter for assessing a radionuclide’s absorbed dose and therapeutic efficacy. In this study, we characterized the *S*-values in a single cell and micrometastatic environment for radionuclides in the ^225^Ac decay chain. The effects of progeny migration and radiopharmaceutical internalization were also assessed.

### Validation of GATE simulations to MIRDcell

Our calculated *S-*values were compared to published values from MIRDcell to validate Geant4-DNA for cellular dosimetry estimates in alpha-emitting radionuclides. We found that for the alpha-emitting radionuclides both the self-dose and cross-dose *S*-values have good agreement with MIRDcell values; however, the *S*-values for the beta emitters showed larger differences from published values (see Tables 1, 2). These larger differences can be accounted for due to the scale of the cells and the range of the low energy beta emissions [53]. This effect has also been seen for electron dose deposition when the size of cell is comparable to the CSDA electron range in liquid water [27]. As the mean beta energy decreased from 660 keV (^209^Tl) to 435 keV (^213^Bi) to 198 keV (^209^Tl), our *S*-values show an underestimation, appropriate estimation, and overestimation, respectively, relative to the MIRDcell *S*-values. For the beta-emitting radionuclides ^131^I, ^90^Y, and ^177^Lu, large per cent differences between MIRDcell and Geant4-DNA *S*-values have been seen, with deviations ranging between − 79 and + 67% [54].

MIRDcell derives *S*-values with an analytic approach using the CSDA to calculate the energy deposition of beta and alpha emissions. However, simulating delta ray production and low energy transport becomes important to accurately estimate cellular dosimetry for low energy beta emissions, and the CSDA is less suitable in these situations where the physical geometry is close to the scale of the range of the beta emissions. There are limitations to the convolution integral method used in MIRD *S*-value calculation at the cellular scale. The CSDA approach neglects the finite range of secondary electrons (δ-rays) and energy loss straggling which are increasingly important on subcellular scales. However, when considering the ^225^Ac chain and its therapeutic applications, the alpha emissions deliver several magnitudes higher doses than the beta emissions (see Fig. 4). Given the strong agreement between the MIRDcell and Geant4-DNA *S*-values for the alpha-emitting isotopes, Geant4-DNA is validated for cellular dosimetry with alpha emitters.

In the MIRD formalism, the geometry of cells is limited to a spherical concentric design which is only an approximation of cancer cell geometries. Different cancer cell lines exhibit different geometries (spherical, ellipsoidal, irregular), which affects cellular *S*-values [20, 55]. Šefl et al. (2015) showed that for Auger-emitting radionuclides the *S*-value differences can be large between spherical and irregular cell geometries [27]. Further investigation is required to determine the magnitude of this effect for alpha-emitting radionuclides. Additionally, the size of the cancer cell and cell nucleus are critical to cellular *S*-values and are very dependent on cancer cell line [56]. Since the overall ^225^Ac decay chain has been well validated with our simplified cellular models, Geant4-DNA simulations would be well suited to further investigate microdosimetry in realistic, complex cellular geometries to evaluate preclinical ^225^Ac radiopharmaceuticals.

### Dose estimates as a function of daughter retention

The primary concern surrounding ^225^Ac-based alpha therapy is the retention of daughter nuclides at the targeted site. While this is an important consideration for toxicity and limiting the injected dose, there is a collateral effect in overestimating absorbed doses to targeted cells. *S-*values to the cell nucleus decrease by up to 72% when there is no retention of ^221^Fr. This scenario is most physically realizable when a radiopharmaceutical is targeting a cell membrane protein and does not undergo internalization into the cell. Similarly, *S*-values decrease by up to 21% when there is no retention of ^213^Bi at the targeted cell. This is a realistic scenario for all source distributions, given that ^213^Bi has a longer half-life of 45 min. Maximizing the retention of progeny radionuclides is actively being researched with liposome carriers [57], polymeric vesicles [58], and nanoparticles [59].

These results highlight the importance of quantifying daughter retention and migration in vivo to ensure accurate absorbed dose measurements are delivered to cancer cells. Progeny migration can be evaluated and quantified in preclinical ^225^Ac radiopharmaceuticals with a variety of methods including alpha-cameras [36, 60], iQID cameras [61, 62], gamma spectroscopy [63], and alpha spectroscopy [64]. With quantified daughter migration, the *S*-values we have reported can be applied to determine the absorbed dose more accurately in preclinical ^225^Ac radiopharmaceutical.

### Effect of cellular internalization of the radiopharmaceutical

We considered only four radionuclide distributions as they are standardized in *S*-values from MIRD. It is noted that cell membrane and cytoplasmic uptake of radioligands are the most physically realistic situations for targeted radiopharmaceutical distribution. However, as novel alpha emitter radiopharmaceuticals are developed, nuclear internalization may become a more common consideration. Overall, nuclear internalization of a ^225^Ac radiopharmaceutical can result in a twofold increase in the cumulative *S*-value; from 0.167 GyBq^−1^ s^−1^ (cell surface only) to 0.364 GyBq^−1^ s^−1^ (fully internalized). With nuclear internalization, the cumulative *S*-value increases threefold to 0.494 GyBq^−1^ s^−1^.

Considering that ^225^Ac has been described as an in vivo alpha generator for therapy, it is critical to quantify the internalization of ^225^Ac radiopharmaceuticals, and account for it while performing microdosimetry estimates. Specific peptides and antibodies are selected to be targeted by radiopharmaceuticals to maximize cellular internalization. It is expected that internalization will vary with each cell-line and targeting moiety. To determine the absorbed dose for a specific radiopharmaceutical, it is necessary to perform an in vitro internalization assay to determine the component of radiopharmaceutical that is membrane bound versus internalized into the cell [65]. With quantified internalization metrics, our *S*-value results can be applied to determine absorbed dose more accurately with preclinical ^225^Ac radiopharmaceuticals. Given the importance of retaining progeny radionuclides and maximizing absorbed dose to the cell nucleus, cellular internalization must also be a priority for developing alpha-emitting radiopharmaceuticals.

## Conclusion

Monte Carlo simulations in GATE (with Geant4-DNA) are a valuable tool for estimating cellular and micrometastatic absorbed doses. While previous work has been heavily focused on beta emitters, this work illustrates GATE’s applications for microdosimetry with alpha-emitting radionuclides. As ^225^Ac radiopharmaceuticals are developed, these results can help to inform the most optimal cellular targets for maximizing absorbed dose to the nucleus of cancer cells. We have also shown the significance of progeny nuclide retention at the targeted site and cellular internalization of the radiopharmaceutical to optimize therapeutic efficacy. Future work will investigate other promising alpha emitters and more realistic complex cellular geometries.

## Data Availability

The datasets generated and analysed in the current study are available from the corresponding author on reasonable request.

## Abbreviations

RPT: Targeted radiopharmaceutical therapy
LET: Linear energy transfer
MIRD: Medical Internal Radiation Dosimetry
CSDA: Continuous slowing down approximation
MCTS: Monte Carlo track-structure
GATE: Geant4 Application for Tomographic Emission
Geant4: GEometry ANd Tracking
RDM: Radioactive Decay Module
ENSDF: Evaluated nuclear structure data file
